# A comparative analysis on risk communication between international and Chinese literature from the perspective of knowledge domain visualization

**DOI:** 10.1186/s12199-021-00981-x

**Published:** 2021-05-28

**Authors:** Huiling Dong, Qunhong Wu, Yue Pang, Bingyi Wu

**Affiliations:** 1 Department of Public Health, Weifang Medical University, No. 7166 Baotong West Street, Weifang, Shandong 261053 China; 2 Department of Health Management, Harbin Medical University, No. 157, Health Road, Nangang District, 150081 Harbin, China; 3 Department of Management, Weifang Medical University, No. 7166 Baotong West Street, Weifang, 261053 Shandong China

**Keywords:** Risk communication, Risk perception, Research hotspot, Knowledge domains

## Abstract

**Background:**

The outbreak of coronavirus disease (COVID-19) severely damaged and endangered people’s lives at the end of 2019. Risk communication plays an important role in the response to it successfully, which has been appreciated by the World Health Organization. Therefore, a comprehensive analysis of risk communication research is necessary, which can understand current research hotspots and reveal new trends.

**Methods:**

In this study, we collected 1134 international articles from the Web of Science database and 3983 Chinese articles from the China National Knowledge Infrastructure database. Bibliometric and mapping knowledge domain analysis methods were used for temporal distribution analysis, cooperation network analysis, co-word network analysis, and burst detection analysis.

**Results:**

The first article in this field was published by western scholars earlier, while the first Chinese article in 2002. Research institutions mainly come from universities. The USA plays a key role in this field. Chinese scholars had a closer cooperation network, but there was less cooperation among domestic institutions. Risk perception, trust, risk management, and risk information had always been the research hotspots in this academic. Trust, sentiment research, and public risk events were essential directions for the future. There are 25 burst words for international articles, while 11 burst words for Chinese articles from 2000 to 2020.

**Conclusions:**

In summary, both domestic and international researchers are concerned about risk communication, risk perception, trust, and risk information. International research on risk communication is systematic and comprehensive relatively. However, Chinese scholars take severe acute respiratory syndrome as the research background and reviewing foreign knowledge as the research starting point. With the purpose of practical and applied research based on a public emergency, the risk communication research lacks continuity in Chinese academy in the past years.

**Supplementary Information:**

The online version contains supplementary material available at 10.1186/s12199-021-00981-x.

## Background

Since December 2019, the novel coronavirus (COVID-19) has broken out and spread in China, Thailand, Japan, South Korea, the USA, Singapore, and other countries, and has gradually evolved into a global pandemic [1]. As a major crisis in human society, COVID-19 has brought huge challenges to individuals, and over the world [2, 3]. On January 31, 2020, the WHO announced that COVID-19 was listed as a “public health emergency of international concern” and made eight recommendations to China. The first one is to “implement a comprehensive risk communication strategy and inform the public about the evolution of the epidemic, preventive measures regularly.” For major public health emergencies, in addition to emergency response, effective risk communication is an indispensable part [4]. Risk communication is an interactive process of exchanging information and views among individuals, groups, and institutions. It involves multidimensional risk properties and related information which not only directly conveys risk-related information, but also expresses the concerns, opinions, and corresponding responses to the risk events, or national regulations and measures in risk management [5].

The international risk communication research is relatively mature, which frontiers mainly come from the USA. In the mid-1980s, the USA established several basic research and application centers for risk communication. Taking 1986 as boundary, risk communication began to become a compelling research focus [6–8]. However, the research in China started late and is still in the stage of exploration and introduction from abroad. At present, most of the influencing factors were analyzed from the perspective of psychology, combined with several important risk events, such as severe acute respiratory syndrome (SARS), tainted milk powder, H1N1, and influenza pandemics [9–12]. By summarizing the study of risk communication at international and domestic academy, the focus of scholars can be summarized into the following categories: First, the study of risk communication is based on media responsibility and communication strategy [13, 14]. Second, research on risk communication is from the perspective of government emergency management [15, 16]. Third, research on risk communication is based on public perception and media literacy [17, 18], in which the public’s risk awareness is an important factor for effective communication.

In recent years, with the frequent occurrence of public emergencies, domestic and foreign scholars have done a lot of research on risk communication. Yet, there are still some problems that need to be solved. What are the hotspots and trends of risk communication? What are the main research forces of risk communication? What are the similarities and differences in risk communication research between international and Chinese academy? Therefore, it is necessary to sort out the characteristics of the development and explore the hotspots, frontiers, and domain of risk communication research. Under the background, we reviewed the articles on risk communication published in the recent 20 years in international and Chinese journals. Then, we used the method of knowledge map to reveal the research strength, frontier, and development trends in this field [19]. Its advantage is that by collecting many documents in a certain period of time, the key elements are analyzed as nodes to help researchers understand the development process quickly [20, 21]. Research conclusions are useful to help people and institutions to pay more attention to risk communication and provide reference for scholars to understand the current situation and trends of risk communication.

## Methods

### Data sources and retrieval

Data used in this study were divided into two categories: international and Chinese data. The international data was downloaded from the Web of Science Core Collection (WoS). A lot of literature showed that WoS was the largest comprehensive academic information resource [21–23]. In our research, WoS included Social Sciences Citation Index (SSCI), Science Citation Index Expanded (SCI-E), and Arts & Humanities Citation Index (A&HCI) databases. The Chinese data was collected from China National Knowledge Infrastructure (CNKI). It has the largest Chinese journal full-text database, including many Chinese journals relating to risk communication [24]. Based on the above points, we chose “risk communication” as keywords. Then, the international data retrieval strategies were set as (TS= (“risk communication”) AND LANGUAGE: (English) AND DOCUMENT TYPES: (Article) Timespan=2000.1.1-2020.12.31. When retrieving Chinese data, we choose “risk communication” as the theme-word, Timespan= “2000-2020”. A total of 4030 articles were retained from WoS, and 1220 articles were retained from CNKI.

After discussing with the team members, we further selected the articles based on inclusion and exclusion criteria to ensure that all the data were closely aligned to the research targets. To ensure the accuracy of sufficient data, in addition to removing repeated articles, we further screened articles according to research purposes. The exclusion criteria are as follows: (1) proceedings paper, interviews, newspaper, book chapter; (2) statistical probability, guidelines on action, disease treatment, medical record management. Finally, 3983 international articles and 1134 Chinese articles were accepted for the analysis after data filtering and removing duplications.

### Data analysis tools

We used CiteSpace 5.5. R2 and Microsoft Excel 2010 for the data analysis. CiteSpace is a document visualization analysis software, which has been widely used in scientific meteorological analysis in various scientific fields [25–27]. CiteSpace 5.5.R2 has different parameter settings and options, resulting in differences in the result indicators [28, 29]. The parameters of this research were set as follows: time slicing (2000–2020), years per slice (1), term source (title, abstract, descriptors, identifiers), node type (author, institution, country, keyword), selection criteria (top 50), and visualization (cluster view-static, show merged network). The corresponding other settings were selected according to different research questions. Microsoft Excel 2010 was used for temporal distribution and polynomial prediction of the number of articles. It should be noted that the results of the CNKI database made by CiteSpace were presented in Chinese. To make it easier to read, we translated the Chinese results into English.

### Data analysis strategies

This study mainly used cooperation network analysis (including authors, institutions, countries network analysis) and co-word network analysis (including keyword co-occurrence network and burst detection analysis). Cooperation network analysis was used to analyze the contribution to different authors, institutions, and countries in one field. In a research cooperation network, the size of a node represents the number of articles [30–32]. Co-word analysis is a content analysis technique that is effective against mapping co-occurrence relationships and the strength of the relationship between a pair of items existing on the same text, revealing the inner construction of a research field. Keyword burst detection analysis can clearly grasp the articles that receive particular attention on the related scientific communities in a certain period. Therefore, the frontiers founded by the burst detection analysis can provide researchers with up-to-date information [32]. The principles of the above analysis methods are mainly compared based on the index of betweenness centrality and burst weight. Betweenness centrality is an index to measure the importance of nodes in the network. Nodes in the purple circle represent documents with betweenness centrality no less than 0.1, which means the authors, institutions, or countries occupied an important position in this field [33]. The burst weight refers to the frequency of a word in a specific time. The higher the weight, the more representative the research trend in that period [34].

## Results

### Sample distribution analysis

**Fig. 1:**
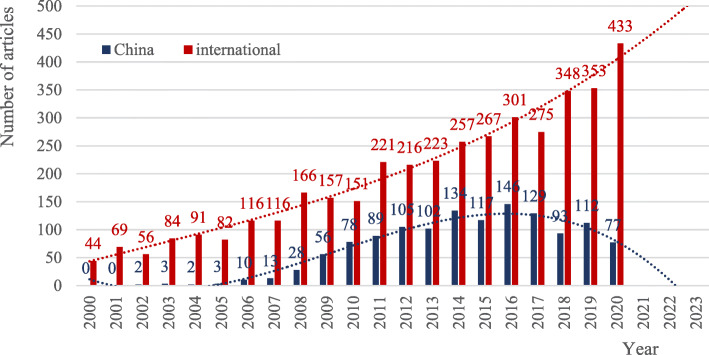
Temporal distribution of international and Chinese risk communication research

Figure 1 illustrates the distribution and trend of articles of international and Chinese scholars. The international research on risk communication started earlier, especially after the 911 incident in 2001 (69), which rose volatility in the following years, with less than 100 article publications before 2006. After that, the number of articles entered a period of rapid rise, reached its peak (433) till 2020. The first Chinese article in this domain was published in 2002. The number of articles was less than 10 before 2006, and increased to 105 in 2012, reached the peak period that followed in 2016 (146), then declined after 2016, with 77 in 2020.

After the descriptive analysis of the data, we conducted a polynomial predictive analysis of the article number in the following 3 years. The results show that international publications increase year by year. In the next 3 years, the number of articles published will increase annually in the world. However, Chinese publications will continue to decrease in the next 3 years. Besides, the gap between Chinese and the international publications will gradually widen.

### Cooperation network analysis

#### Co-author

**Fig. 2:**
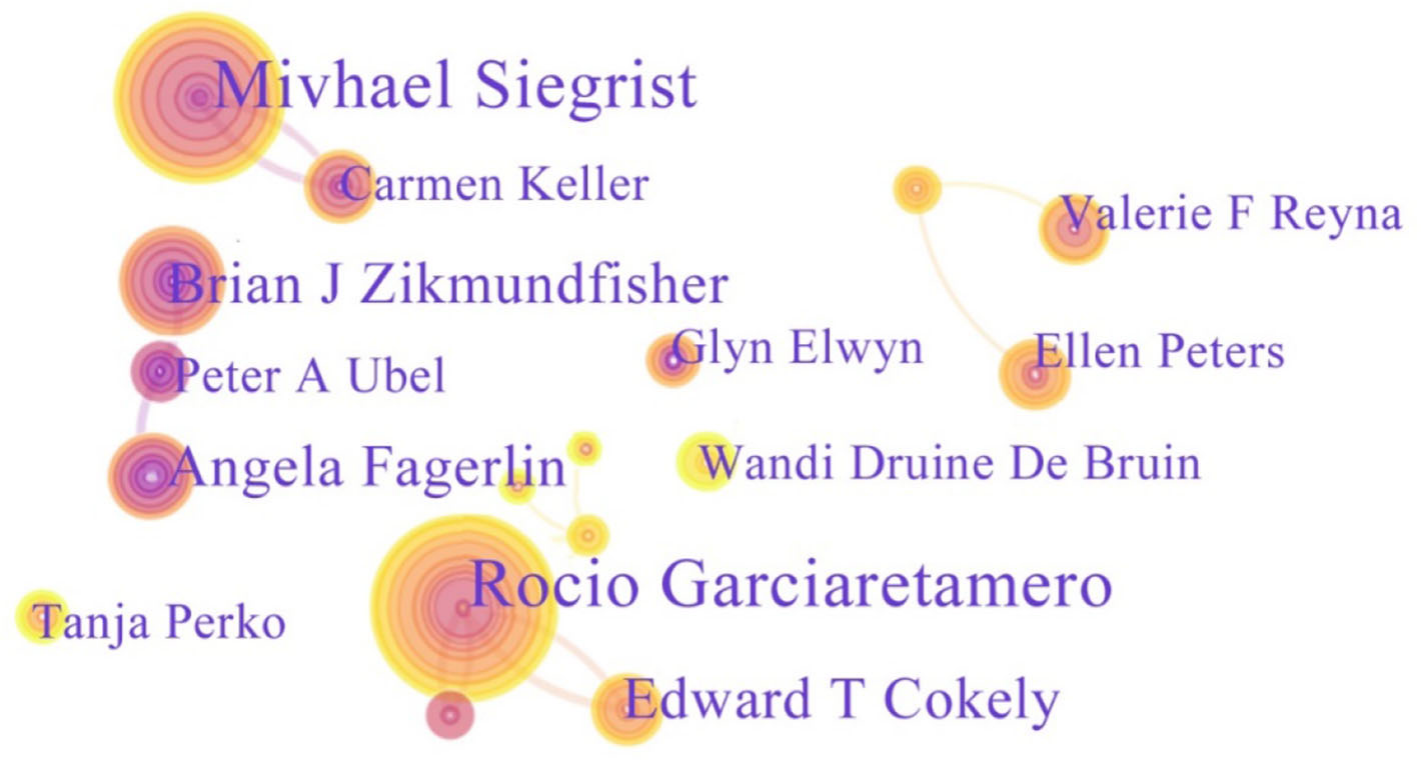
Co-author network of international database

**Fig. 3:**
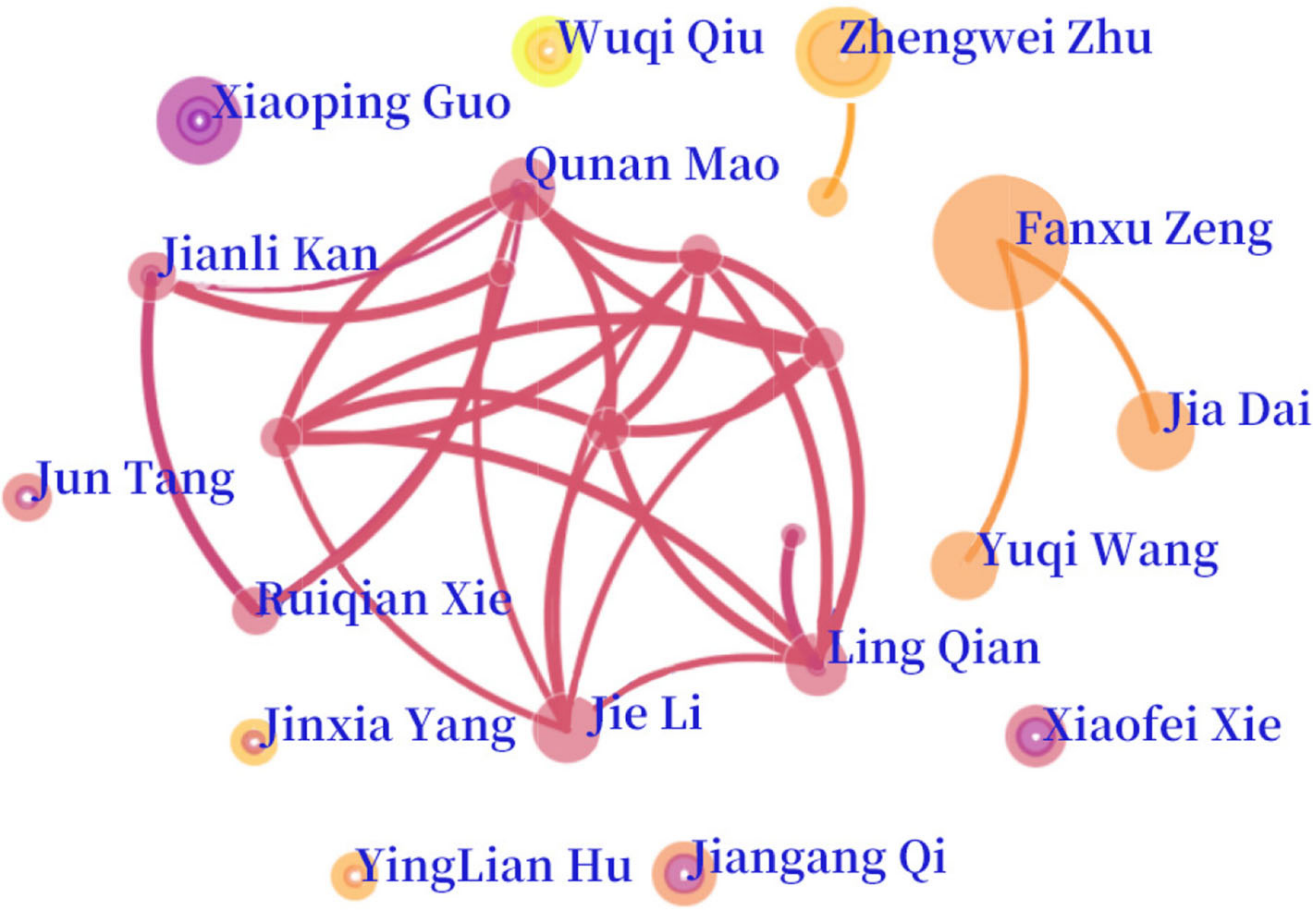
Co-author network of Chinese database

Figures 2 and 3 show the analysis of international and Chinese authors’ cooperation networks, indicating that many authors have conducted research on risk communication, and some scholars were collaborators. Internationally, Rocio Garcia-Retamero had the highest number of articles with 31, next was Michael Siegrist with 28, followed by Brian J. Zikmund-Fisher, Angela Fagerlin, and Valerie F Reyna with 18, 14, and 12, respectively, which showed that these authors were very concerned about this field. In China, Fanxu Zeng had the highest number of articles (9), followed by Zhengwei Zhu and Xiaoping Guo, both with 7 articles. Wuqi Qiu and Jia Dai both had published more than 6 articles. The collaborator network diagram showed that many research teams had studied risk communication research, but domestic and international cooperation research was not close enough. Relatively speaking, the cooperation network of domestic scholars was closer, and foreign researchers were more independent.

#### Co-institution

Just like the co-author, many institutions have researched on risk communication. Internationally, eight of the top 10 research institutions were universities, indicating that universities had published the greatest number of articles (Table 1). The highest number of international university publications was from King’s College London with 73, followed by Cornell University, Harvard University, and University of Michigan and Washington University with more than 48. The highest centrality ranking was Cardiff University (0.20), followed by Harvard University (0.17), University of Michigan (0.16), and Washington University (0.16), which indicates institutions had higher publication quality.

**Table 1:** The volume and centrality of domestic and international risk communication institutions

International institutions	Count	Centrality	Chinese institutions	Count	Centrality
King’s College London	73	0.11	Chinese Health Education Center	14	0.00
Cornell University	68	0.06	School of Public Policy and Management, Xian Jiaotong University	11	0.00
Harvard University	57	0.17	School of Journalism and Communication, Tsinghua University	10	0.00
University of Michigan	48	0.16	School of Law, Zhongnan University of Economics and Law	8	0.00
University of Washington	48	0.16	School of Journalism and Communication, Huazhong University of Science and Technology	6	0.00
University of Pennsylvania	44	0.06	School of Public Administration, Tsinghua University	6	0.00
Max Planck Institute for Human Development	43	0.01	School of Communication, East China Normal University	5	0.00
Ctr Dis Control Prevent	42	0.02	National School of Administration	4	0.00
Cardiff University	41	0.20	Chinese Center for Disease Control and Prevention	4	0.00
The University of Sydney	41	0.04	School of Political Science and Public Administration, Shandong University	4	0.00

In China, the organization publishing the largest number of articles was the Chinese Health Education Center (14), indicating that the organization had published the largest number of articles, which was a bit different from international ones, followed by the School of Public Policy and Management of Xian Jiaotong University, School of Journalism and Communication of Tsinghua University, and School of Law of Zhongnan University of Economics and Law with more than 8 articles published. The centrality of all institutions in China was 0, which meant that the influence of articles published by Chinese institutions had not reached the leading level. The network diagram of the publication number showed that the network of cooperation among international institutions was closer than that of Chinese institutions.

#### Co-country

Table 2 summarizes the top 10 countries in terms of volume and centrality of international risk communication research. We can see that the development of risk communication research varied among countries. Geographically, half of them were concentrated in Europe. Among these countries, the USA was the most productive country, far ahead of other countries in this field, in which centrality was also the largest (0.25). It showed that the USA not only had conducted the highest publication quantity, but also had more advanced than others. Although China ranked seventh on this list, its central position was almost 0, which showed that Chinese scholars had not published articles appreciated enough internationally. It was worth noting that although the number of articles in New Zealand (269) and Switzerland (122) was much lower than that in the USA (1791), the centrality of articles in these two countries was more than 0.10, which showed that the quality of articles was still high.

**Table 2:** Top 10 countries in the published volume and centrality of international database.

Country	Count	Centrality
USA	1791	0.25
England	513	0.15
Canada	286	0.11
Germany	284	0.11
New Zealand	269	0.21
Australia	218	0.00
People’s Republic of China	152	0.00
Italy	137	0.08
Japan	132	0.00
Switzerland	122	0.17

### Co-word network analysis

#### Keyword co-occurrence network analysis

**Fig. 4:**
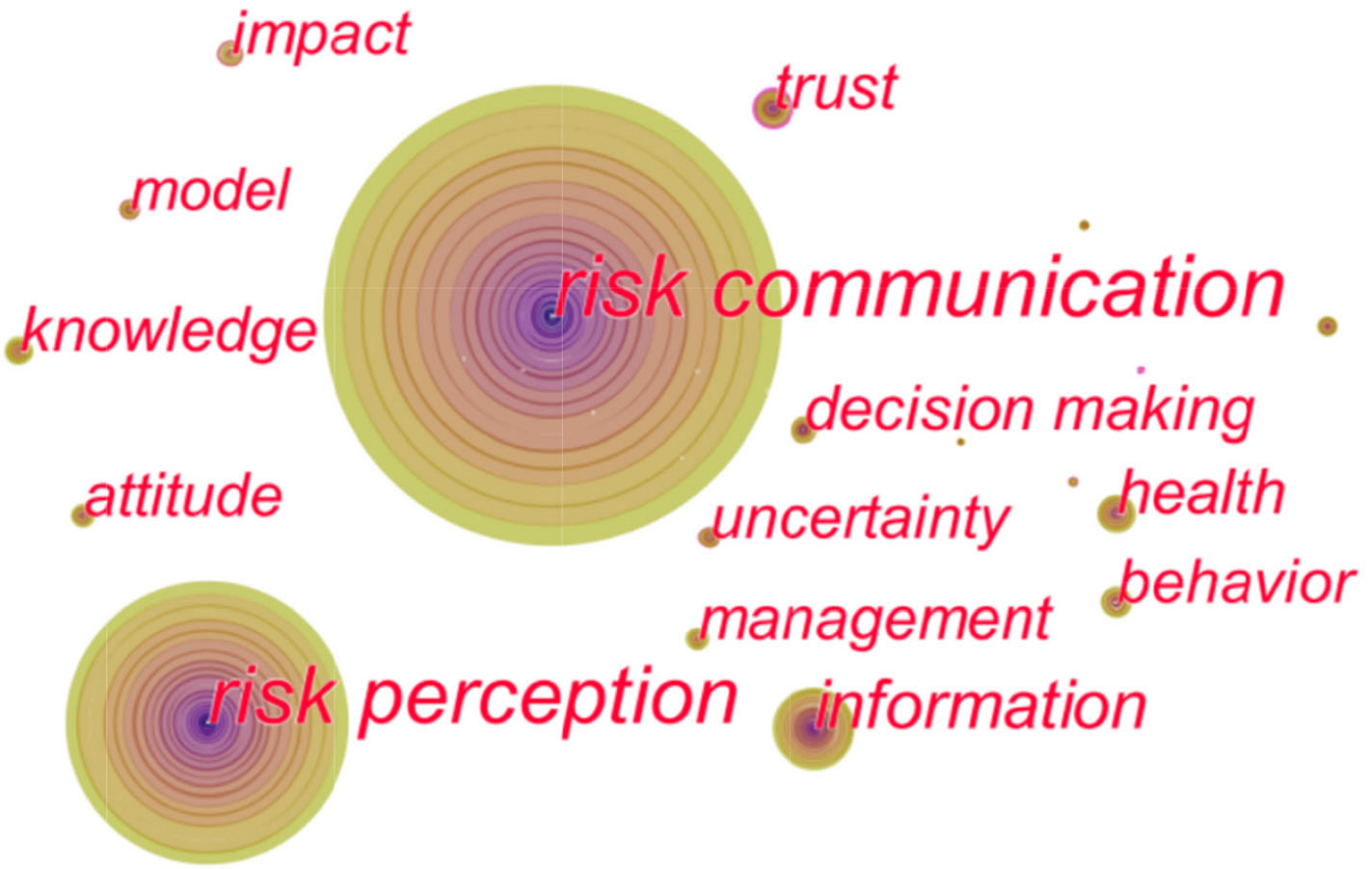
Keyword co-occurrence network of international database

The research hotspots can be explored by analyzing the keywords in the literature. In WoS, the top 10 important keywords were extracted after merging keywords with the same expression content, which were risk communication (2333), risk perception (1496), information (517), health (296), trust (293), knowledge (266), decision making (256), behavior (243), management (242), and impact (240). Among all keywords, the top three betweenness centrality were trust (0.37), risk communication (0.19), and risk perception (0.18), which means that they have greater influence than other keywords, as shown in Fig. 4. It can be seen from the citation frequency and centrality of the above keywords that “trust” had the largest intermediary centrality, indicating that trust was a key issue in risk communication research. The citation frequency of risk communication had a prominent position, far higher than other keywords. The research of risk communication based on risk perception had always been the focus of the public and decision-makers.

**Fig. 5:**
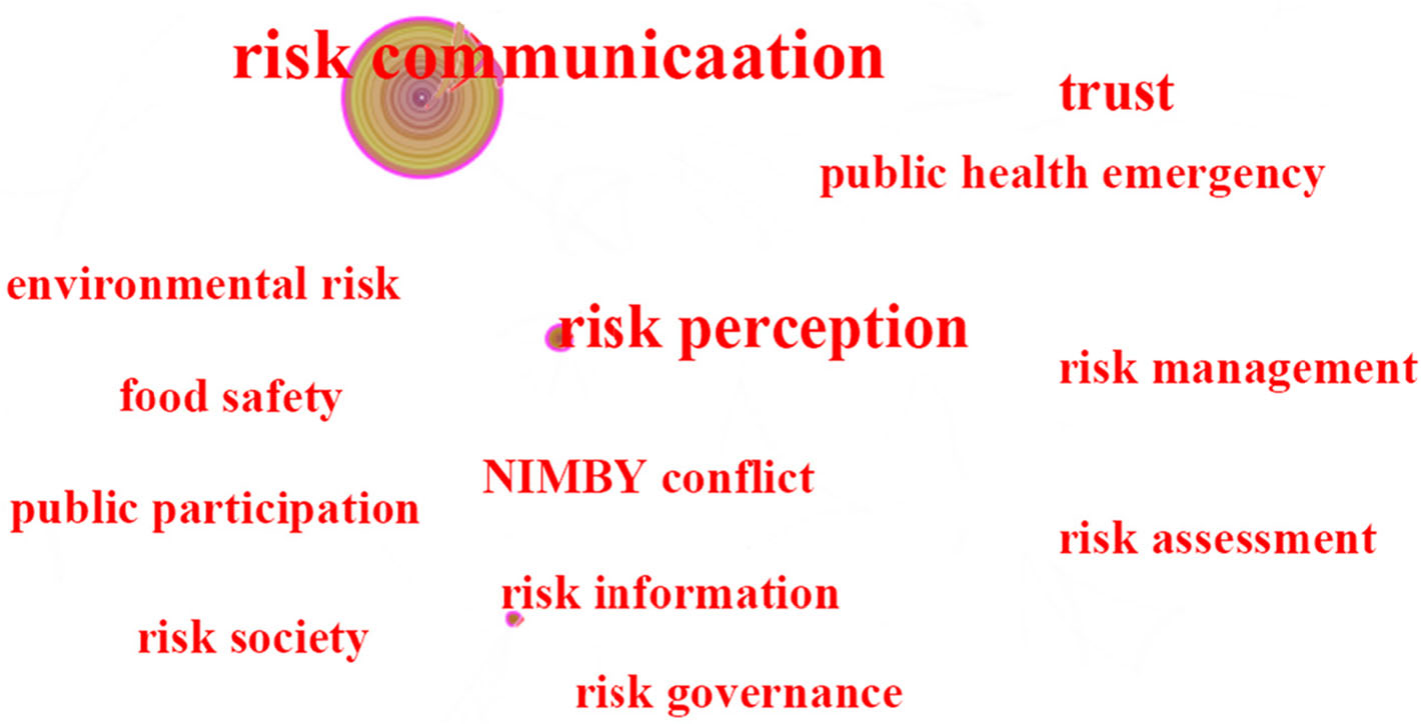
Keyword co-occurrence network of Chinese database

In China, the frequency of the top 10 keywords was risk communication (457), risk perception (119), risk information (81), risk management (71), risk society (52), public participation (36), food safety (30), “Not In My Backyard” (NIMBY) conflicts (28), public health emergencies (27), and trust (26) (Fig. 5). The top centrality ranks were food safety (0.65), risk communication (0.54), risk information (0.49), risk perception (0.49), and trust (0.25). Consistent with international research, the citation frequency of risk communication and risk perception is much higher than other keywords, and the centrality was also higher. Secondly, risk management, risk society, and risk communication appeared more frequently, which provided the basis for related risk communication research. The three keywords of food safety (30), NIMBY conflicts (28), and public health emergency (27) indicated that most of Chinese risk communication research was based on one specific emergency. In addition, the betweenness centrality of risk communication (0.54) and trust (0.25) was also very high, indicating that “trust” has become the key research point that affected the risk communication.

**Fig. 6:**
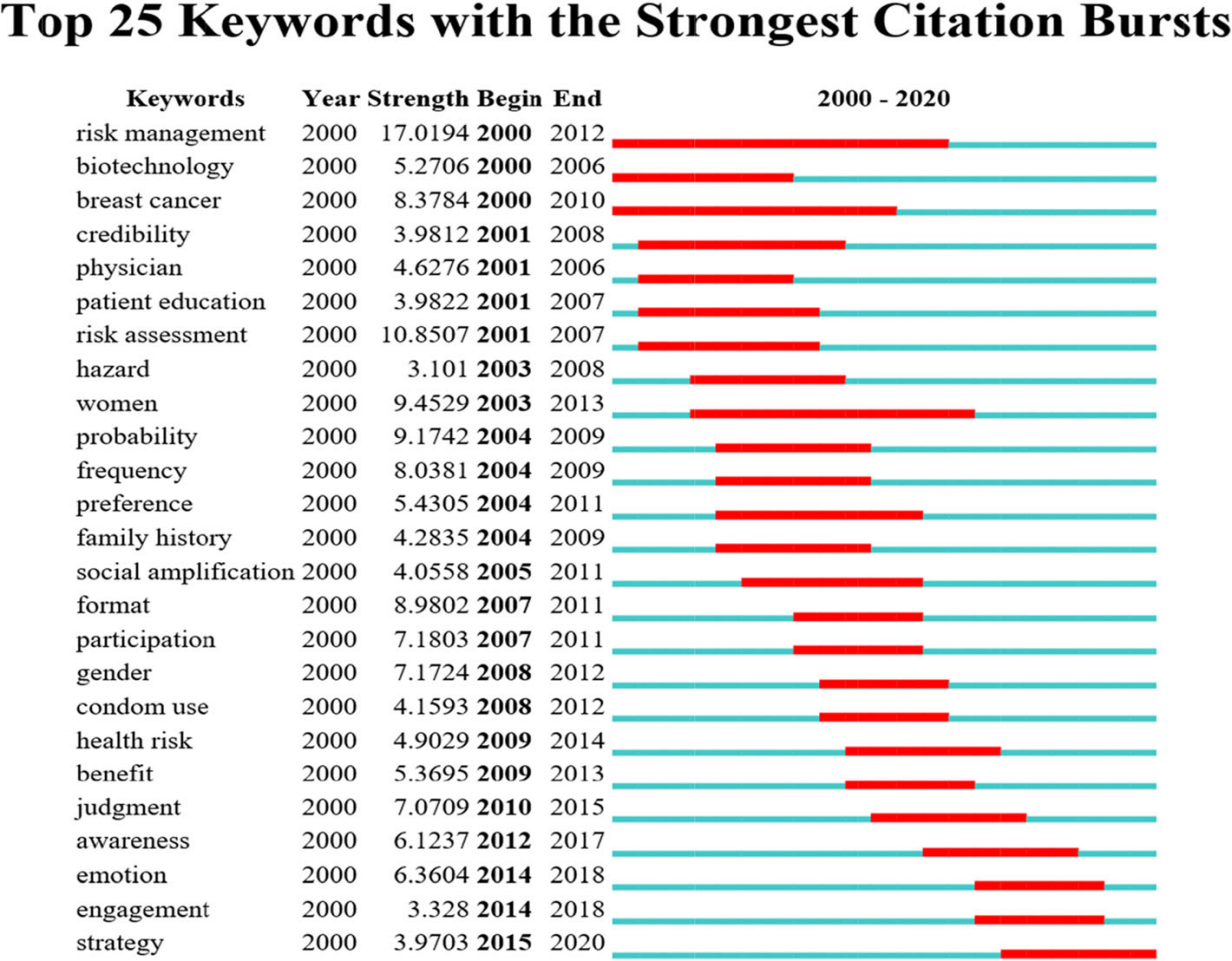
Keywords with the strongest citation bursts of international database

**Fig. 7:**
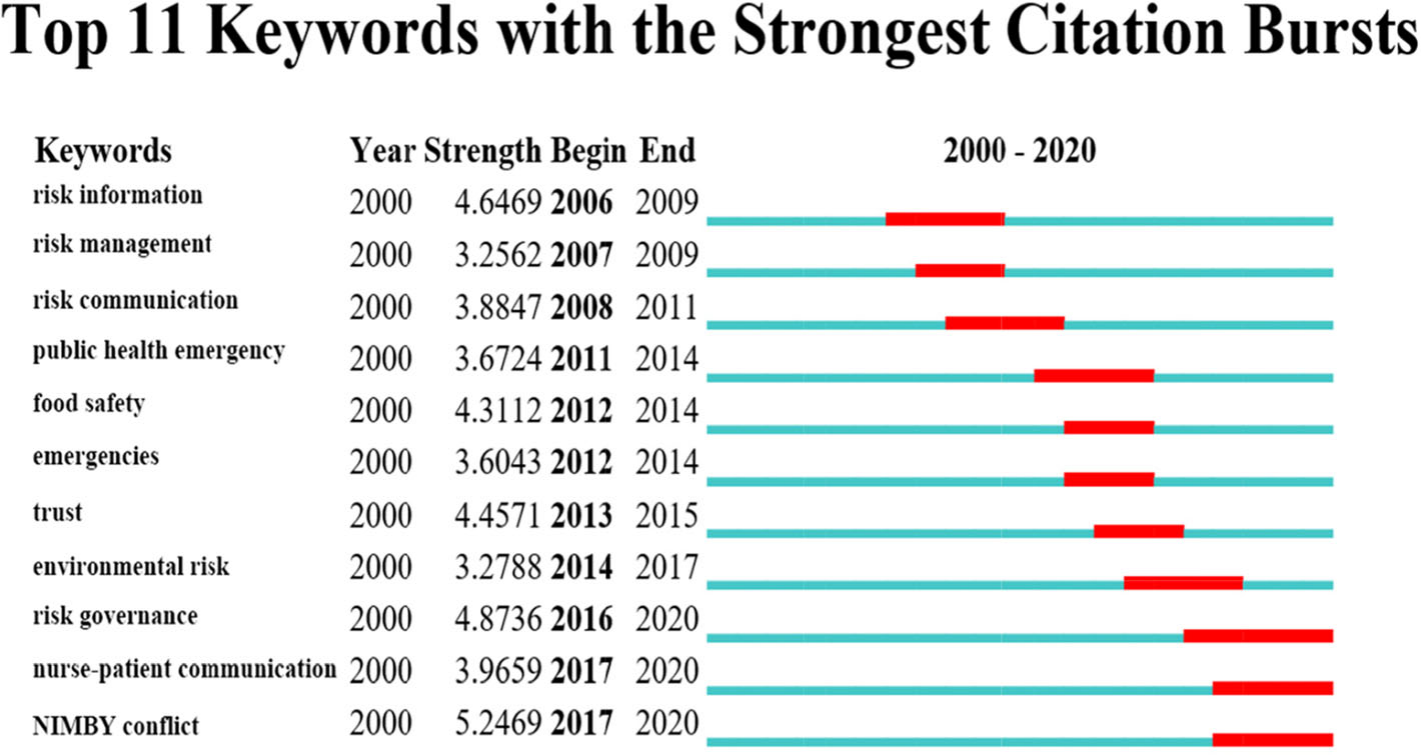
Keywords with the strongest citation bursts of Chinese database

Figures 6 and 7 illustrate the keywords with the strongest citation burst for international and Chinese database. A total of 25 burst words appeared in the international field, while the Chinese data detected burst words with 11. International researches can be divided into four stages. The first stage was 2000–2007, in which the mutation weights of risk management and risk assessment were higher, indicating that more attention was paid to these two studies in this time period. The second stage was 2008–2011. At this time, the frequency and form of risk communication received special attention, and public participation and women had become hot topics. The third stage was 2012–2017. The weight of burst in decision-making and public awareness was higher, which had become a new trend in this time. The fourth stage was 2018–2020. Emotion research and strategy research had entered the view of researchers. The weight of burst was higher, which had become a new research hotspot.

Chinese risk communication research was also divided into three phases. The first phase was from 2006 to 2011. During this period, research on risk communication, risk management, and risk information was fundamental research. The second stage was 2012–2017. Trust and various public emergencies (public health emergencies, food safety, emergencies, environmental risks) had become the research hotspot during this period. The third stage was 2018–2020. Nurse-patient communication and NIMBY incidents had become the focus topics for researchers.

## Discussion

We have found out that international research on risk communication is earlier than in China. International scholars pay more and more attention to risk communication, which has been growing over time. From the perspective of article number, international risk communication research has been increasing with time since 2000, reaching a peak in 2020, which is associated with the COVID-19 outbreak and is expected to continue to grow in the next 3 years. The occurrence of risk communication in the 10 years from 2009 to 2019 showed a general trend of first rising and then slowly declining. It is likely related to the number of significant events that occur in each year [35]. The peak number of articles was reached in 2016, which may be related to the frequent occurrence of vaccine safety events, food safety incidents, and NIMBY conflict events (In China, “NIMBY conflict” refers to the strong, resolute, and highly emotional collective opposition or even protest behavior taken by residents for fear that government construction projects, such as garbage dump, nuclear power plant, funeral home, and other NIMBY facilities could bring about many negative effects on their health and environmental quality.). In the following years, China did not have a large impact of public security emergencies. Research on risk communication has declined due to fewer hot topics. Although the number of Chinese articles will decrease in the next 3 years, it remained above 76. Overall, risk communication is still valued because of its importance in emergency management.

The research scholars and institutions of domestic and international need to strengthen cooperation with each other. The results of this study show that some scholars and research institutions have already cooperated, but the degree of closeness is not enough. Previous research believed that cooperation between different research institutions was very effective in promoting high-level and effective research, helped to develop more mature research areas [36]. Therefore, domestic and international scholars should strengthen close cooperation between different institutions. It is worth noting that the number of articles seems not to be related to the centrality. For example, the international university with the largest number of articles was King’s College London with 73, while the centrality ranked fifth. Therefore, we believe that the influence of the article is not only determined by the number of articles, but also depends on the content and innovative and advanced methods of the article. In addition, the centrality of all institutions in China was 0, which meant that the influence of articles published by Chinese institutions had not reached the leading level. The number of articles published by each institution is small and scattered, which may lead to superficial research, insufficient authority, and lower recognition in China.

Risk cognition is a research hotspot in both domestic and international risk communication research, and it is also the foundation to further research on risk communication. Through the keyword analysis, risk perception is cited at high frequency at domestic and international, and the centrality is relatively larger. Some studies have shown that individuals’ perception of risk plays a leading role in the process of risk communication [37, 38]. However, risk perception is affected by many factors. The randomness and subjectivity generated by personal factors and social factors make the perception of risk difficult to measure [39, 40]. Some research has concluded that there were 15 main factors directly related to risk perception, such as the uncertainty of risks and the sources of risks [41, 42]. But if it is specific to a certain field, such as a public health emergency or public safety incident, are these factors applied to? What variables will affect the public risk perception? What kind of attitudes and measures should the public adopt due to risk perception? These issues need to be studied further by relevant scholars.

“Trust” is a key factor affecting the effectiveness of risk communication. Through the keyword analysis, “trust” ranks among the best in risk communication, and its centrality is relatively high, especially among international researchers (0.74). Besides, “trust” has become an important direction of international researchers from 2003 to 2007 in citation bursts analysis. Domestic researchers in this field paid attention to the influencing factors of “trust” mainly in 2013–2015. The literature research found that the current research can be divided into three aspects: First, the research on the “lack of trust” between the public, experts, and policy makers [42]. Risk perception of the public is based on personal experience, while experts and policy makers proceed from objective risk assessments to weigh risks and benefits [43]. The second is the study of factors affecting trust [44]. The third is the research on the asymmetry of trust construction. Research literature shows that risk communication is based on trust [45]. But trust is often formed gradually in a long-term process. According to the 2016 China Comprehensive Well-off Index [46], food safety ranked first among the top ten industries the public distrusted, followed by pension policies and house prices. The report shows that at present, our country has a low degree of social trust in food safety, and the safety incidents caused by food occur frequently. At the same time, more and more scholars pay attention to the problem of risk communication and trust in food safety. In essence, food safety has been an important research issue in the field of risk communication in China.

In summary, the current Chinese and international research institutes focus on the same topics, focusing on risk communication, risk perception, risk assessment, and risk information. However, due to differences in specific national conditions and risk communication development, domestic and international scholars have different levels of attention to specific issues. Through this research, it is found that domestic research pays more attention to the understanding and improvement of public risk awareness based on health issues, focusing on the trust analysis of audiences and information disseminators. The previous research on risk communication is more systematic and comprehensive. In contrast, sudden public safety events such as H1N1, influenza pandemic, food safety, and NIMBY conflict events have a higher burst weight in domestic research, indicating that risk communication is mainly based on specific emergency occurrence, which may expose the lack of persistence in the research of China.

Admittedly, there are some limitations to this study. The conclusions drawn in this study should be based only on the findings of two large literature databases abovementioned, which is necessary to meet literature demand for the study. Since the current version of CiteSpace cannot achieve a better combination of synonymous keywords, it is necessary to merge keywords manually, which can easily affect the objectivity of the results. In addition, this article aims to analyze the development of risk communication research and find its characteristics and hotspots in the academic field. Therefore, we could not deeply analyze some specific links of risk communication, such as risk communication mechanisms, risk monitoring, risk assessment, and other issues.

## Conclusions

According to the analyses, conclusions are as follows. In the next 3 years, the number of international risk communication articles will continue to increase, while that of China will decline. The degree of cooperation between Chinese scholars is closer than that of international scholars, yet there is less cooperation among different organizations in China. At present, researchers both at domestic and international are concerned about risk communication, risk perception, trust, and risk information. Due to differences in specific national conditions and risk communication development, scholars tended to have different levels of attention to specific issues. The international research on risk communication is more systematic and comprehensive. Chinese researchers take SARS as the research starting point, foreign knowledge review as research background, which purpose is to propose practical and applied counter-measures about one kind of public health event. Therefore, risk communication research in China inevitably lacks continuity in this domain.

## Supplementary Information

**Additional file 1: Table S1.** Top 10 authors in the published volume and centrality of international database. **Table S2.** Top 10 authors in the published volume and centrality of Chinese database. **Table S3.** Top 10 keywords ranked by citation counts and centrality of international database. **Table S4.** Top 10 keywords ranked by citation counts and centrality of Chinese database. **Figure S1.** Co-author network of Chinese database. **Figure S2.** Co-institution network of International database.

## Data Availability

All data generated or analyzed during this study are included in this published article and its supplementary information files.

## Abbreviations

NIMBY: Not In My Backyard
COVID-19: Coronavirus disease 2019
WoS: Web of Science
SCI-E: Science Citation Index Expanded
SSCI: Social Sciences Citation Index
A&HCI: Arts & Humanities Citation Index
CNKI: China National Knowledge Infrastructure
SARS: Severe acute respiratory syndrome
