# Supplementary material for: A comparative analysis on risk communication between international and Chinese literature from the perspective of knowledge domain visualization
Source: Environ Health Prev Med. 2021 May 28;26:60. doi: 10.1186/s12199-021-00981-x (PMC8162189; doi:10.1186/s12199-021-00981-x)
Supplement: Supplementary file 1 — Additional file 1: Table S1. Top 10 authors in the published volume and centrality of international database. Table S2. Top 10 authors in the published volume and centrality of Chinese database. Table S3. Top 10 keywords ranked by citation counts and centrality of international database. Table S4. Top 10 keywords ranked by citation counts and centrality of Chinese database. Figure S1. Co-author network of Chinese database. Figure S2. Co-institution network of International database. [file 12199_2021_981_MOESM1_ESM.docx]

**A Comparative Study of International and Chinese Risk Communication from the Perspective of Knowledge Domains Visualization**

| **Table S1. Top 10 authors in the published volume and centrality of international database.** | | | |
| --- | --- | --- | --- |
| Number | Author | Count | Centrality |
| 1 | ROCIO GARCIARETAMERO | 31 | 0.05 |
| 2 | MICHAEI SIEGRIST | 28 | 0.02 |
| 3 | BRIAN J.ZIKMUNDFISHER | 18 | 0.01 |
| 4 | ANGELA FAGERLIN | 14 | 0.00 |
| 5 | Valerie F REYNA | 12 | 0.00 |
| 6 | ELLLEN PETERS | 12 | 0.00 |
| 7 | EDWARD T COKELY | 12 | 0.00 |
| 8 | CARMEN KELLER | 10 | 0.00 |
| 9 | A EDWARDS | 10 | 0.00 |
| 10 | ANN BOSTROM | 10 | 0.00 |

| **Table S2. Top 10 authors in the published volume and centrality of Chinese database** | | | |
| --- | --- | --- | --- |
| Number | 作者 | 发文量 | 中心性 |
| 1 | Zhengwei Zhu(朱正威) | 9 | 0.01 |
| 2 | Fanxu Zeng(曾繁旭) | 9 | 0.00 |
| 3 | Xiaoping Guo(郭小平) | 7 | 0.00 |
| 4 | Wuqi Qiu(邱五七) | 6 | 0.00 |
| 5 | Jia Dai(戴佳) | 6 | 0.00 |
| 6 | Qunan Mao(毛群安) | 5 | 0.01 |
| 7 | Ling Qian(钱玲) | 5 | 0.00 |
| 8 | Ruiqian Xie(解瑞谦) | 5 | 0.00 |
| 9 | Jianli Yan(阎坚力) | 5 | 0.00 |
| 10 | Jie Li(李杰) | 5 | 0.00 |

| **Table S3. Top 10 keywords ranked by citation counts and centrality of international database** | | | |
| --- | --- | --- | --- |
| Number | **Keyword** | **Count** | **Centrality** |
| 1 | risk communication | 2333 | 0.19 |
| 2 | risk perception | 1496 | 0.18 |
| 3 | information | 517 | 0.02 |
| 4 | health | 296 | 0.05 |
| 5 | trust | 293 | 0.37 |
| 6 | knowledge | 266 | 0.02 |
| 7 | decision making | 251 | 0.09 |
| 8 | behavior | 243 | 0.02 |
| 9 | management | 242 | 0.04 |
| 10 | impact | 240 | 0.08 |

**Table S4. Top 10 keywords ranked by citation counts and centrality of Chinese database.**

| Number | 作者 | 发文量 | 中心性 |
| --- | --- | --- | --- |
| 1 | risk communication (风险沟通) | 457 | 0.49 |
| 2 | risk perception (风险认知) | 119 | 0.49 |
| 3 | risk information (风险信息) | 81 | 0.54 |
| 4 | risk management (风险管理) | 71 | 0.33 |
| 5 | risk society (风险社会) | 52 | 0.27 |
| 6 | public participation (公众参与) | 36 | 0.05 |
| 7 | food safety (食品安全) | 30 | 0.65 |
| 8 | NIMBY conflicts (邻避冲突) | 28 | 0.1 |
| 9 | public health emergencies (突发公共  卫生事件) | 27 | 0.16 |
| 10 | Trust (信任) | 26 | 0.25 |


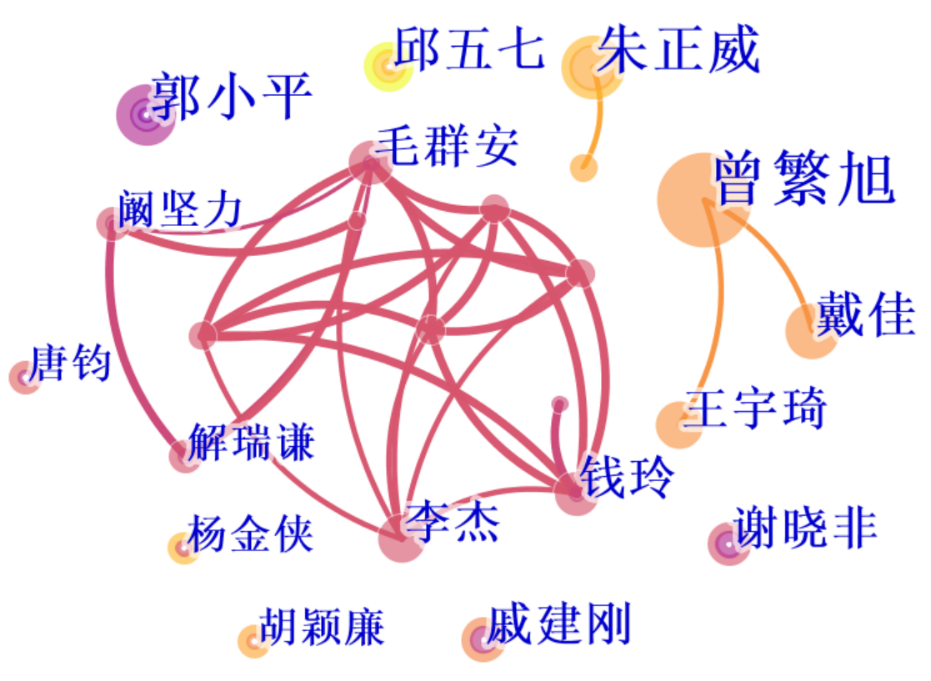


**Figure S1. Co-author network of Chinese database**


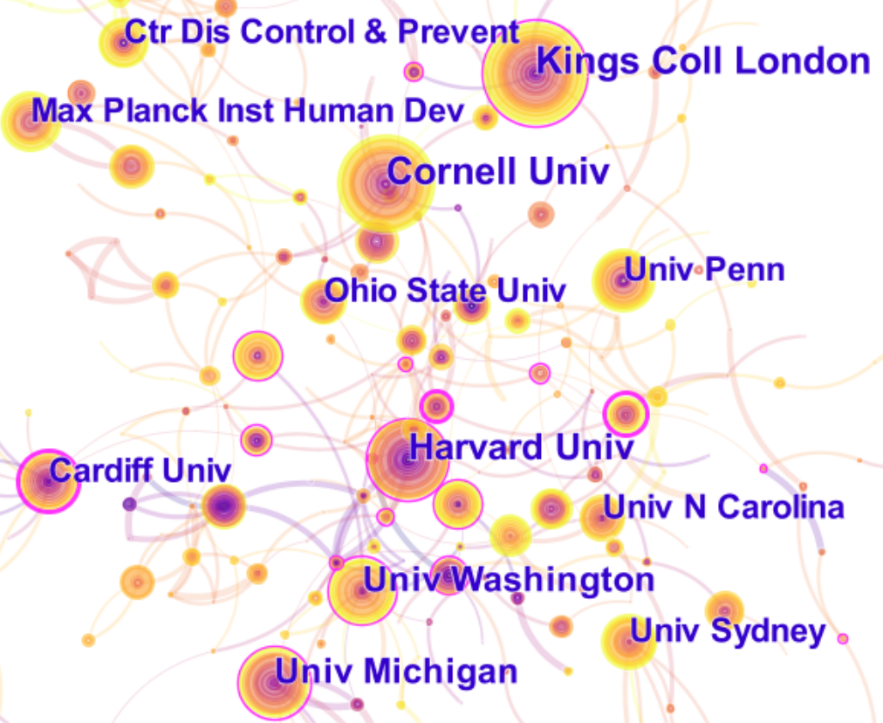


**Figure S2. Co-institution network of International database.**
